# 1,5-Dimethyl-2-phenyl-1*H*-pyrazol-3(2*H*)-one–4,4′-(propane-2,2-di­yl)bis­[1,5-dimethyl-2-phenyl-1*H*-pyrazol-3(2*H*)-one] (1/1)

**DOI:** 10.1107/S160053681205091X

**Published:** 2012-12-22

**Authors:** Krzysztof Lyczko

**Affiliations:** a Institute of Nuclear Chemistry and Technology, Dorodna 16, 03-195 Warsaw, Poland

## Abstract

The asymmetric unit of the title compound, C_11_H_12_N_2_O·C_25_H_28_N_4_O_2_, contains two different mol­ecules. The smaller is known as anti­pyrine [systematic name: 1,5-dimethyl-2-phenyl-1*H*-pyrazol-3(2*H*)-one] and the larger is built up from two antypirine mol­ecules which are connected through a C atom of the pyrazolone ring to a central propanyl part [systematic name: 4,4′-(propane-2,2-di­yl)bis­[1,5-dimethyl-2-phenyl-1*H*-pyrazol-3(2*H*)-one]. Intra­molecular C—H⋯O hydrogen bonds occur in the latter mol­ecule. In the crystal, C—H⋯O hydrogen bonds link the mol­ecules into a two-dimensional network parallel to (001).

## Related literature

Structural data on metal complexes with anti­pyrine were reported by Vijayan & Viswamitra (1966 ▶); Biagini Cingi *et al.* (1972 ▶); Baker & Jeffery (1974 ▶); Brassy *et al.* (1974 ▶); Mahadevan *et al.* (1984 ▶); Rheingold & King (1989 ▶) and Su *et al.* (2000 ▶). For related structures, see: Singh & Vijayan (1973 ▶); Panneerselvam *et al.* (1996 ▶); Merz (2002 ▶); Yuchi *et al.* (1991 ▶). Some properties of anti­pyrine and its derivatives were described by Peter *et al.* (1991 ▶).
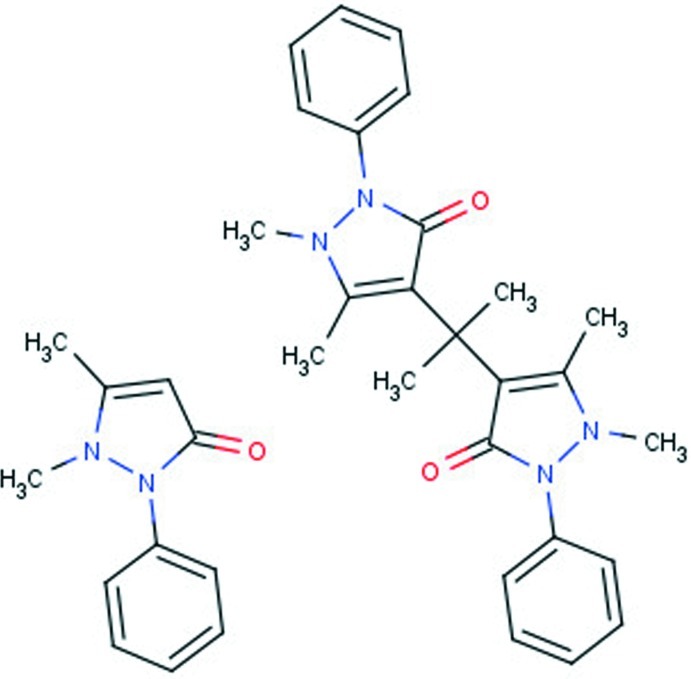


## Experimental

### 

#### Crystal data

C_11_H_12_N_2_O·C_25_H_28_N_4_O_2_
*M* *_r_* = 604.74
Monoclinic, 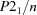

*a* = 11.1751 (3) Å
*b* = 7.4623 (2) Å
*c* = 37.2830 (8) Å
β = 91.570 (2)°
*V* = 3107.93 (14) Å^3^
*Z* = 4
Cu *K*α radiation
μ = 0.67 mm^−1^
*T* = 100 K
0.30 × 0.25 × 0.15 mm

#### Data collection

Agilent SuperNova (Dual, Cu at zero, Eos) diffractometer
Absorption correction: multi-scan (*CrysAlis PRO*; Agilent, 2010 ▶) *T*_min_ = 0.938, *T*_max_ = 1.000
12009 measured reflections
6009 independent reflections
5366 reflections with *I* > 2σ(*I*)
*R*_int_ = 0.018

#### Refinement

*R*[*F* ^2^ > 2σ(*F* ^2^)] = 0.040
*wR*(*F* ^2^) = 0.099
*S* = 1.03
6009 reflections
414 parameters
H-atom parameters constrained
Δρ_max_ = 0.26 e Å^−3^
Δρ_min_ = −0.31 e Å^−3^

### 

Data collection: *CrysAlis PRO* (Agilent, 2010 ▶); cell refinement: *CrysAlis PRO*; data reduction: *CrysAlis PRO*; program(s) used to solve structure: *SHELXS97* (Sheldrick, 2008 ▶); program(s) used to refine structure: *SHELXL97* (Sheldrick, 2008 ▶); molecular graphics: *XP* in *SHELXTL* (Sheldrick, 2008 ▶) and *Mercury* (Macrae *et al.*, 2008 ▶); software used to prepare material for publication: *SHELXL97*.

## Supplementary Material

Crystal structure: contains datablock(s) global, I. DOI: 10.1107/S160053681205091X/kj2216sup1.cif

Structure factors: contains datablock(s) I. DOI: 10.1107/S160053681205091X/kj2216Isup2.hkl

Supplementary material file. DOI: 10.1107/S160053681205091X/kj2216Isup3.cml

Additional supplementary materials:  crystallographic information; 3D view; checkCIF report

## Figures and Tables

**Table 1:** Hydrogen-bond geometry (Å, °)

*D*—H⋯*A*	*D*—H	H⋯*A*	*D*⋯*A*	*D*—H⋯*A*
C2—H2⋯O1^i^	0.95	2.50	3.2949 (17)	142
C5—H5*C*⋯O2	0.98	2.44	3.3930 (17)	163
C10—H10⋯O3^ii^	0.95	2.53	3.4670 (18)	171
C14—H14*A*⋯O3	0.98	2.45	3.1228 (17)	126
C14—H14*B*⋯O2	0.98	2.34	3.0275 (17)	126
C21—H21⋯O1^iii^	0.95	2.49	3.3428 (18)	150
C25—H25⋯O2	0.95	2.37	2.8811 (17)	113
C29—H29*B*⋯O3^iv^	0.98	2.38	3.2956 (17)	155
C30—H30*A*⋯O3^iv^	0.98	2.32	3.3015 (16)	176

Symmetry codes: (i) 
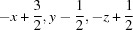
; (ii) 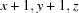; (iii) 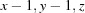; (iv) 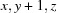.

**Fig. 1.**
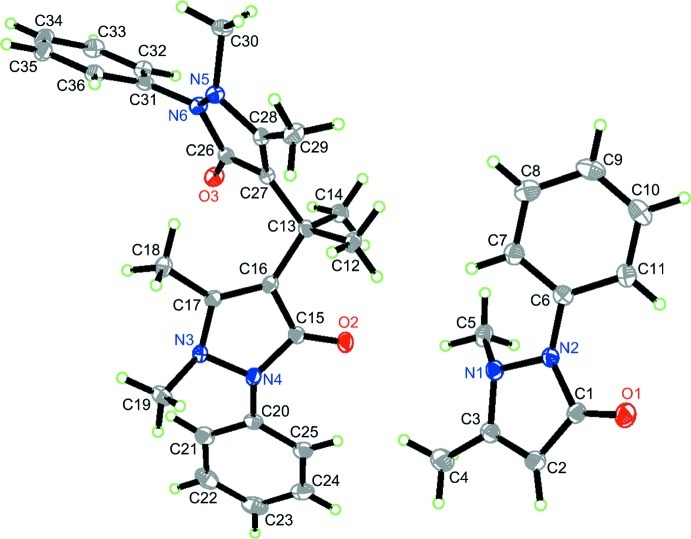
A molecular structure of the title compounds. Displacement ellipsoids of the non-hydrogen atoms are drawn at the 50% probability level.

**Fig. 2.**
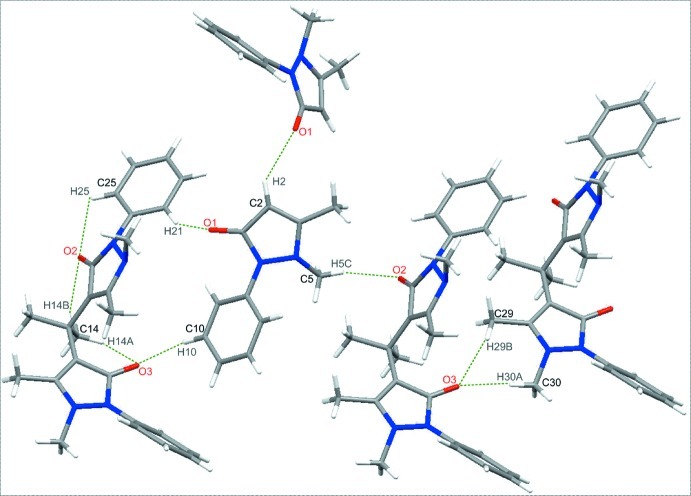
A fragment of the crystal structure showing the intra- and intermolecular hydrogen bonds.

## supplementary crystallographic information

### Comment

1,5-Dimethyl-2-phenyl-1H-pyrazol-3(2H)-one, known as antipyrine or phenazone, is an interesting synthetic compound from the medicinal (pharmaceutical) point of view because of its analgesic and antipyretic properties. It is also used as a probe of oxidative metabolism (Peter *et al.*, 1991). Antipyrine is a bulky monodentate donor ligand. Its functional carbonyl group can coordinate to metal ions. Some structures of homoleptic antipyrine-metal complexes were punlished, such as [Pb(antipyrine)_6_](ClO_4_)_2_ (Vijayan & Viswamitra, 1966), [Y(antipyrine)_6_]I_3_ (Baker & Jeffery, 1974), [Cd(antipyrine)_6_](ClO_4_)_2_ (Mahadevan *et al.*, 1984), [Tb(antipyrine)_6_]I_3_ (Rheingold & King, 1989) and [Tb(antipyrine)_6_](ClO_4_)_3_ (Su *et al.*, 2000). Moreover, the crystal structures of other complexes, *e.g.*, [Zn(antipyrine)_2_Cl_2_] (Biagini Cingi *et al.*, 1972) and [Cu(antipyrine)_2_(NO_3_)_2_] (Brassy *et al.*, 1974) have been reported.

The aim of this work was to crystallize the hexa-coordinated complex between Pb^II^ ions and antipyrine with nitrate as a counterion. Previously the hexakis(antipyrine)lead(II) perchlorate complex was successfully crystallized from a solution in water (Vijayan & Viswamitra, 1966). Unexpectedly, under the reaction conditions applied in this work (see Experimental), instead of the hexakis(antipyrine)lead(II) nitrate complex, two different organic molecules were co-crystallized (Fig. 1). The first one is antipyrine and the second one is 4,4'-propane-2,2-diyldiantipyrine - a compound containing two antipyrine molecules linked by a propanyl group. The molecules in the crystal structure are interacting together through very weak intermolecular C—H···O hydrogen bonds (Table 1, Fig. 2) forming a two-dimensional network parallel to (0 0 1). Additionally, the structure of 4,4'-propane-2,2-diyldiantipyrine is stabilized by three intramolecular C—H···O hydrogen bonds (Table 1, Fig. 2).

Previously antipyrine (Singh & Vijayan, 1973), 4-hydroxyantipyrine (Panneerselvam *et al.*, 1996) and 4,4'-methylenediantipyrine (Merz, 2002), the similar compounds to 4,4'-propane-2,2-diyldiantipyrine, were crystallized. The reaction of 4,4'-methylenediantipyrine with titanium(IV) has been used for selective photometric determination of this cation. The structure of tris(4,4'-methylenediantipyrine)titanium(IV) perchlorate complex has been presented by Yuchi *et al.*, (1991).

The presence of an antipyrine derivative in this crystal structure is extremely strange and in my opinion can probably be ascribed to the catalyzed conversion of antipyrine into such a complicated compound as in the presented reaction system.

### Experimental

The title compounds were crystallized unintentionally in an attempt to synthesize single crystals of the hexakis(antipyrine)lead(II) nitrate complex. Lead(II) nitrate (0.236 g, 0.712 mmol) and antipyrine (0.809 g, 4.293 mmol) were dissolved in 1.0 ml of water and methanol (1:1). Next about 1/5 of the solvent was evaporated off at a temperature of about 75°C. After one year of storage in the refrigerator some colourless block like crystals were found.

### Refinement

H atoms were placed in calculated positions with C—H = 0.98 (methyl) or 0.95 Å (aromatic) and were refined isotropically using a riding model with *U*_iso_(H) = 1.5 *U*_eq_(C) for methyl H atoms and *U*_iso_(H) = 1.2 *U*_eq_(C) for aromatic H atoms.

### Crystal data

**Table:**

C_25_H_28_N_4_O_2_·C_11_H_12_N_2_O	*F*(000) = 1288
*M**_r_* = 604.74	*D*_x_ = 1.292 Mg m^−^^3^
Monoclinic, *P*2_1_/*n*	Cu *K*α radiation, λ = 1.54178 Å
Hall symbol: -P 2yn	Cell parameters from 5594 reflections
*a* = 11.1751 (3) Å	θ = 3.6–71.8°
*b* = 7.4623 (2) Å	µ = 0.67 mm^−^^1^
*c* = 37.2830 (8) Å	*T* = 100 K
β = 91.570 (2)°	Block, colourless
*V* = 3107.93 (14) Å^3^	0.30 × 0.25 × 0.15 mm
*Z* = 4	

### Data collection

**Table:**

Agilent SuperNova (Dual, Cu at zero, Eos) diffractometer	6009 independent reflections
Radiation source: SuperNova (Cu) X-ray Source	5366 reflections with *I* > 2σ(*I*)
Mirror monochromator	*R*_int_ = 0.018
Detector resolution: 16.0131 pixels mm^-1^	θ_max_ = 72.0°, θ_min_ = 4.1°
ω scans	*h* = −13→13
Absorption correction: multi-scan (*CrysAlis PRO*; Agilent, 2010)	*k* = −4→9
*T*_min_ = 0.938, *T*_max_ = 1.000	*l* = −45→42
12009 measured reflections	

### Refinement

**Table:**

Refinement on *F*^2^	Primary atom site location: structure-invariant direct methods
Least-squares matrix: full	Secondary atom site location: difference Fourier map
*R*[*F*^2^ > 2σ(*F*^2^)] = 0.040	Hydrogen site location: inferred from neighbouring sites
*wR*(*F*^2^) = 0.099	H-atom parameters constrained
*S* = 1.03	*w* = 1/[σ^2^(*F*_o_^2^) + (0.0422*P*)^2^ + 1.6129*P*] where *P* = (*F*_o_^2^ + 2*F*_c_^2^)/3
6009 reflections	(Δ/σ)_max_ < 0.001
414 parameters	Δρ_max_ = 0.26 e Å^−^^3^
0 restraints	Δρ_min_ = −0.31 e Å^−^^3^

### Special details

**Table:**

Geometry. All e.s.d.'s (except the e.s.d. in the dihedral angle between two l.s. planes) are estimated using the full covariance matrix. The cell e.s.d.'s are taken into account individually in the estimation of e.s.d.'s in distances, angles and torsion angles; correlations between e.s.d.'s in cell parameters are only used when they are defined by crystal symmetry. An approximate (isotropic) treatment of cell e.s.d.'s is used for estimating e.s.d.'s involving l.s. planes.
Refinement. Refinement of *F*^2^ against ALL reflections. The weighted *R*-factor *wR* and goodness of fit *S* are based on *F*^2^, conventional *R*-factors *R* are based on *F*, with *F* set to zero for negative *F*^2^. The threshold expression of *F*^2^ > σ(*F*^2^) is used only for calculating *R*-factors(gt) *etc*. and is not relevant to the choice of reflections for refinement. *R*-factors based on *F*^2^ are statistically about twice as large as those based on *F*, and *R*- factors based on ALL data will be even larger.

### Fractional atomic coordinates and isotropic or equivalent isotropic displacement parameters (Å^2^)

**Table:**

	*x*	*y*	*z*	*U*_iso_*/*U*_eq_	
C1	0.73885 (12)	0.5494 (2)	0.19813 (4)	0.0176 (3)	
C2	0.70121 (13)	0.4021 (2)	0.22001 (4)	0.0201 (3)	
H2	0.7288	0.3775	0.2438	0.024*	
C3	0.61974 (12)	0.30441 (19)	0.20087 (4)	0.0191 (3)	
C4	0.55955 (15)	0.1333 (2)	0.21067 (4)	0.0281 (3)	
H4A	0.5944	0.0341	0.1973	0.042*	
H4B	0.4738	0.1418	0.2047	0.042*	
H4C	0.5710	0.1117	0.2365	0.042*	
C5	0.57888 (13)	0.2704 (2)	0.13580 (4)	0.0201 (3)	
H5A	0.6543	0.2098	0.1306	0.030*	
H5B	0.5546	0.3459	0.1154	0.030*	
H5C	0.5167	0.1808	0.1400	0.030*	
C6	0.65888 (12)	0.65797 (18)	0.13835 (3)	0.0160 (3)	
C7	0.54464 (13)	0.69605 (19)	0.12467 (4)	0.0191 (3)	
H7	0.4769	0.6379	0.1342	0.023*	
C8	0.53013 (14)	0.8194 (2)	0.09705 (4)	0.0230 (3)	
H8	0.4524	0.8436	0.0873	0.028*	
C9	0.62883 (15)	0.9074 (2)	0.08366 (4)	0.0246 (3)	
H9	0.6188	0.9905	0.0645	0.029*	
C10	0.74213 (14)	0.87403 (19)	0.09827 (4)	0.0227 (3)	
H10	0.8091	0.9375	0.0896	0.027*	
C11	0.75846 (13)	0.74826 (19)	0.12551 (4)	0.0189 (3)	
H11	0.8362	0.7241	0.1352	0.023*	
N1	0.59546 (10)	0.38236 (16)	0.16795 (3)	0.0166 (2)	
N2	0.67665 (10)	0.52572 (16)	0.16531 (3)	0.0161 (2)	
O1	0.80765 (9)	0.67590 (15)	0.20421 (3)	0.0244 (2)	
C12	0.26030 (12)	0.43381 (19)	0.11446 (4)	0.0179 (3)	
H12A	0.2893	0.5128	0.0956	0.027*	
H12B	0.3282	0.3712	0.1259	0.027*	
H12C	0.2192	0.5053	0.1324	0.027*	
C13	0.17264 (11)	0.29586 (17)	0.09789 (3)	0.0139 (3)	
C14	0.24097 (12)	0.18958 (19)	0.06911 (4)	0.0182 (3)	
H14A	0.1884	0.0971	0.0587	0.027*	
H14B	0.3118	0.1329	0.0803	0.027*	
H14C	0.2659	0.2716	0.0502	0.027*	
C15	0.22095 (12)	0.04833 (18)	0.14514 (3)	0.0154 (3)	
C16	0.13547 (11)	0.17061 (17)	0.12818 (3)	0.0137 (3)	
C17	0.03074 (12)	0.15149 (18)	0.14544 (3)	0.0143 (3)	
C18	−0.08452 (12)	0.2527 (2)	0.14323 (4)	0.0201 (3)	
H18A	−0.0899	0.3321	0.1641	0.030*	
H18B	−0.1516	0.1682	0.1430	0.030*	
H18C	−0.0876	0.3244	0.1212	0.030*	
C19	−0.01645 (13)	0.0347 (2)	0.20569 (4)	0.0209 (3)	
H19A	0.0201	0.1378	0.2180	0.031*	
H19B	−0.0029	−0.0736	0.2201	0.031*	
H19C	−0.1027	0.0550	0.2024	0.031*	
C20	0.19083 (12)	−0.20882 (18)	0.18707 (3)	0.0163 (3)	
C21	0.10387 (13)	−0.3368 (2)	0.19420 (4)	0.0205 (3)	
H21	0.0220	−0.3141	0.1883	0.025*	
C22	0.13810 (15)	−0.4980 (2)	0.21008 (4)	0.0267 (3)	
H22	0.0786	−0.5836	0.2158	0.032*	
C23	0.25744 (15)	−0.5362 (2)	0.21774 (4)	0.0279 (3)	
H23	0.2800	−0.6475	0.2283	0.034*	
C24	0.34341 (14)	−0.4095 (2)	0.20980 (4)	0.0248 (3)	
H24	0.4255	−0.4356	0.2145	0.030*	
C25	0.31133 (13)	−0.2447 (2)	0.19507 (3)	0.0192 (3)	
H25	0.3708	−0.1573	0.1905	0.023*	
C26	−0.02192 (11)	0.26949 (18)	0.05944 (3)	0.0137 (3)	
C27	0.06521 (11)	0.38047 (18)	0.07869 (3)	0.0135 (3)	
C28	0.02835 (11)	0.55412 (18)	0.07508 (3)	0.0138 (3)	
C29	0.08036 (13)	0.72665 (18)	0.08890 (4)	0.0189 (3)	
H29A	0.0926	0.7190	0.1150	0.028*	
H29B	0.0252	0.8252	0.0831	0.028*	
H29C	0.1573	0.7486	0.0777	0.028*	
C30	−0.10277 (12)	0.69931 (18)	0.02819 (3)	0.0169 (3)	
H30A	−0.0781	0.8171	0.0373	0.025*	
H30B	−0.1883	0.7018	0.0217	0.025*	
H30C	−0.0568	0.6698	0.0070	0.025*	
C31	−0.22286 (12)	0.34094 (18)	0.03238 (3)	0.0143 (3)	
C32	−0.23650 (13)	0.20851 (19)	0.00621 (4)	0.0176 (3)	
H32	−0.1684	0.1509	−0.0032	0.021*	
C33	−0.35122 (13)	0.16203 (19)	−0.00586 (4)	0.0204 (3)	
H33	−0.3616	0.0719	−0.0237	0.024*	
C34	−0.45076 (13)	0.2464 (2)	0.00795 (4)	0.0217 (3)	
H34	−0.5288	0.2144	−0.0005	0.026*	
C35	−0.43591 (13)	0.37752 (19)	0.03412 (4)	0.0199 (3)	
H35	−0.5040	0.4350	0.0436	0.024*	
C36	−0.32209 (12)	0.42511 (19)	0.04652 (3)	0.0170 (3)	
H36	−0.3120	0.5144	0.0645	0.020*	
N3	0.03776 (10)	0.01203 (16)	0.17043 (3)	0.0154 (2)	
N4	0.15874 (10)	−0.04046 (16)	0.17199 (3)	0.0160 (2)	
N5	−0.08054 (10)	0.56315 (14)	0.05609 (3)	0.0137 (2)	
N6	−0.10503 (10)	0.38756 (15)	0.04440 (3)	0.0142 (2)	
O2	0.32678 (8)	0.01806 (14)	0.13877 (3)	0.0215 (2)	
O3	−0.03072 (9)	0.10492 (12)	0.05679 (3)	0.0181 (2)	

### Atomic displacement parameters (Å^2^)

**Table:**

	*U*^11^	*U*^22^	*U*^33^	*U*^12^	*U*^13^	*U*^23^
C1	0.0138 (6)	0.0231 (7)	0.0160 (6)	0.0009 (6)	0.0012 (5)	−0.0033 (5)
C2	0.0194 (7)	0.0249 (7)	0.0161 (6)	0.0009 (6)	0.0015 (5)	0.0000 (6)
C3	0.0176 (7)	0.0194 (7)	0.0204 (7)	0.0026 (6)	0.0028 (5)	0.0013 (5)
C4	0.0316 (8)	0.0238 (8)	0.0290 (8)	−0.0051 (7)	0.0001 (6)	0.0059 (6)
C5	0.0169 (7)	0.0216 (7)	0.0218 (7)	−0.0013 (6)	−0.0005 (5)	−0.0064 (6)
C6	0.0189 (7)	0.0148 (6)	0.0142 (6)	0.0003 (5)	0.0023 (5)	−0.0031 (5)
C7	0.0184 (7)	0.0203 (7)	0.0187 (6)	0.0008 (6)	0.0028 (5)	−0.0022 (5)
C8	0.0255 (8)	0.0229 (7)	0.0204 (7)	0.0082 (6)	−0.0002 (6)	−0.0022 (6)
C9	0.0395 (9)	0.0160 (7)	0.0184 (7)	0.0049 (6)	0.0045 (6)	0.0006 (6)
C10	0.0303 (8)	0.0162 (7)	0.0219 (7)	−0.0042 (6)	0.0093 (6)	−0.0041 (6)
C11	0.0191 (7)	0.0186 (7)	0.0193 (6)	−0.0010 (6)	0.0042 (5)	−0.0045 (5)
N1	0.0166 (6)	0.0151 (6)	0.0180 (5)	−0.0034 (5)	0.0004 (4)	−0.0011 (4)
N2	0.0150 (5)	0.0169 (6)	0.0162 (5)	−0.0033 (5)	−0.0002 (4)	0.0000 (4)
O1	0.0228 (5)	0.0292 (6)	0.0210 (5)	−0.0091 (5)	−0.0016 (4)	−0.0043 (4)
C12	0.0142 (6)	0.0164 (7)	0.0228 (7)	−0.0012 (5)	−0.0019 (5)	0.0007 (5)
C13	0.0128 (6)	0.0128 (6)	0.0161 (6)	−0.0007 (5)	0.0010 (5)	0.0002 (5)
C14	0.0174 (7)	0.0194 (7)	0.0180 (6)	0.0027 (6)	0.0029 (5)	0.0009 (5)
C15	0.0152 (6)	0.0139 (6)	0.0173 (6)	−0.0012 (5)	0.0005 (5)	0.0005 (5)
C16	0.0130 (6)	0.0123 (6)	0.0157 (6)	−0.0003 (5)	−0.0008 (5)	−0.0016 (5)
C17	0.0141 (6)	0.0147 (6)	0.0141 (6)	−0.0004 (5)	−0.0017 (5)	−0.0011 (5)
C18	0.0146 (7)	0.0243 (7)	0.0216 (7)	0.0044 (6)	0.0026 (5)	0.0007 (6)
C19	0.0245 (7)	0.0214 (7)	0.0171 (7)	0.0008 (6)	0.0062 (5)	0.0010 (5)
C20	0.0213 (7)	0.0147 (6)	0.0128 (6)	0.0009 (5)	0.0007 (5)	−0.0001 (5)
C21	0.0227 (7)	0.0203 (7)	0.0184 (7)	−0.0015 (6)	0.0017 (5)	−0.0003 (5)
C22	0.0382 (9)	0.0179 (7)	0.0242 (7)	−0.0038 (7)	0.0067 (6)	0.0026 (6)
C23	0.0435 (10)	0.0187 (7)	0.0218 (7)	0.0084 (7)	0.0039 (6)	0.0053 (6)
C24	0.0293 (8)	0.0279 (8)	0.0170 (7)	0.0104 (7)	−0.0005 (6)	0.0014 (6)
C25	0.0214 (7)	0.0209 (7)	0.0152 (6)	0.0012 (6)	−0.0006 (5)	0.0000 (5)
C26	0.0141 (6)	0.0138 (6)	0.0133 (6)	0.0003 (5)	0.0019 (5)	−0.0005 (5)
C27	0.0133 (6)	0.0135 (6)	0.0138 (6)	−0.0009 (5)	0.0020 (5)	−0.0007 (5)
C28	0.0129 (6)	0.0146 (6)	0.0140 (6)	−0.0012 (5)	0.0013 (5)	−0.0009 (5)
C29	0.0207 (7)	0.0119 (6)	0.0239 (7)	0.0001 (5)	−0.0048 (5)	−0.0016 (5)
C30	0.0207 (7)	0.0141 (6)	0.0159 (6)	0.0008 (5)	−0.0004 (5)	0.0023 (5)
C31	0.0159 (6)	0.0130 (6)	0.0138 (6)	−0.0021 (5)	−0.0018 (5)	0.0024 (5)
C32	0.0201 (7)	0.0157 (7)	0.0168 (6)	0.0004 (5)	0.0005 (5)	−0.0008 (5)
C33	0.0246 (7)	0.0174 (7)	0.0188 (6)	−0.0026 (6)	−0.0041 (5)	−0.0020 (5)
C34	0.0180 (7)	0.0223 (7)	0.0246 (7)	−0.0043 (6)	−0.0060 (5)	0.0030 (6)
C35	0.0165 (7)	0.0193 (7)	0.0239 (7)	0.0014 (6)	0.0009 (5)	0.0022 (6)
C36	0.0197 (7)	0.0158 (7)	0.0156 (6)	−0.0006 (5)	0.0002 (5)	−0.0005 (5)
N3	0.0116 (5)	0.0184 (6)	0.0162 (5)	0.0011 (4)	0.0017 (4)	0.0012 (4)
N4	0.0110 (5)	0.0175 (6)	0.0196 (6)	0.0004 (4)	0.0003 (4)	0.0025 (5)
N5	0.0162 (5)	0.0095 (5)	0.0154 (5)	−0.0006 (4)	−0.0011 (4)	−0.0007 (4)
N6	0.0154 (6)	0.0100 (5)	0.0170 (5)	−0.0006 (4)	−0.0016 (4)	−0.0012 (4)
O2	0.0123 (5)	0.0237 (5)	0.0287 (5)	0.0033 (4)	0.0038 (4)	0.0073 (4)
O3	0.0193 (5)	0.0114 (5)	0.0236 (5)	−0.0008 (4)	−0.0027 (4)	−0.0009 (4)

### Geometric parameters (Å, º)

**Table:**

C1—C2	1.439 (2)	C19—H19C	0.9800
C2—H2	0.9500	C20—C21	1.393 (2)
C3—C2	1.354 (2)	C20—C25	1.3972 (19)
C3—C4	1.493 (2)	C21—H21	0.9500
C4—H4A	0.9800	C22—C21	1.390 (2)
C4—H4B	0.9800	C22—C23	1.386 (2)
C4—H4C	0.9800	C22—H22	0.9500
C5—H5A	0.9800	C23—H23	0.9500
C5—H5B	0.9800	C24—C23	1.386 (2)
C5—H5C	0.9800	C24—C25	1.390 (2)
C6—C7	1.3909 (19)	C24—H24	0.9500
C7—H7	0.9500	C25—H25	0.9500
C8—C7	1.387 (2)	C27—C26	1.4529 (18)
C8—C9	1.389 (2)	C28—C27	1.3652 (19)
C8—H8	0.9500	C28—C29	1.4982 (18)
C9—H9	0.9500	C29—H29A	0.9800
C10—C9	1.387 (2)	C29—H29B	0.9800
C10—H10	0.9500	C29—H29C	0.9800
C11—C6	1.3966 (19)	C30—H30A	0.9800
C11—C10	1.391 (2)	C30—H30B	0.9800
C11—H11	0.9500	C30—H30C	0.9800
N1—C3	1.3785 (18)	C31—C32	1.3942 (18)
N1—C5	1.4683 (17)	C32—H32	0.9500
N2—C1	1.4018 (17)	C33—C32	1.391 (2)
N2—C6	1.4187 (17)	C33—C34	1.389 (2)
N2—N1	1.4079 (16)	C33—H33	0.9500
O1—C1	1.2342 (17)	C34—H34	0.9500
C12—H12A	0.9800	C35—C34	1.389 (2)
C12—H12B	0.9800	C35—H35	0.9500
C12—H12C	0.9800	C36—C31	1.3908 (19)
C13—C12	1.5388 (18)	C36—C35	1.3877 (19)
C13—C16	1.5318 (17)	C36—H36	0.9500
C13—C27	1.5185 (18)	N3—C17	1.3975 (17)
C14—C13	1.5519 (18)	N3—C19	1.4724 (16)
C14—H14A	0.9800	N4—C15	1.4009 (17)
C14—H14B	0.9800	N4—C20	1.4184 (17)
C14—H14C	0.9800	N4—N3	1.4072 (15)
C15—C16	1.4533 (18)	O2—C15	1.2335 (16)
C17—C16	1.3587 (18)	O3—C26	1.2357 (17)
C17—C18	1.4936 (18)	N5—C28	1.3926 (17)
C18—H18A	0.9800	N5—C30	1.4705 (16)
C18—H18B	0.9800	N5—N6	1.4054 (15)
C18—H18C	0.9800	N6—C26	1.3873 (17)
C19—H19A	0.9800	N6—C31	1.4225 (17)
C19—H19B	0.9800		
			
O1—C1—C2	132.09 (13)	H18B—C18—H18C	109.5
O1—C1—N2	123.27 (13)	H19A—C19—H19B	109.5
N2—C1—C2	104.63 (12)	H19A—C19—H19C	109.5
C3—C2—C1	108.39 (12)	H19B—C19—H19C	109.5
C3—C2—H2	125.8	N3—C19—H19A	109.5
C1—C2—H2	125.8	N3—C19—H19B	109.5
C2—C3—C4	129.21 (13)	N3—C19—H19C	109.5
C2—C3—N1	110.80 (13)	C21—C20—C25	120.07 (13)
N1—C3—C4	119.98 (13)	C21—C20—N4	120.84 (12)
C3—C4—H4A	109.5	C25—C20—N4	119.09 (12)
C3—C4—H4B	109.5	C20—C21—H21	120.3
C3—C4—H4C	109.5	C22—C21—C20	119.30 (14)
H4A—C4—H4B	109.5	C22—C21—H21	120.3
H4A—C4—H4C	109.5	C21—C22—H22	119.4
H4B—C4—H4C	109.5	C23—C22—C21	121.17 (14)
H5A—C5—H5B	109.5	C23—C22—H22	119.4
H5A—C5—H5C	109.5	C22—C23—H23	120.5
H5B—C5—H5C	109.5	C24—C23—C22	119.00 (14)
N1—C5—H5A	109.5	C24—C23—H23	120.5
N1—C5—H5B	109.5	C23—C24—C25	121.01 (14)
N1—C5—H5C	109.5	C23—C24—H24	119.5
C7—C6—C11	120.51 (13)	C25—C24—H24	119.5
C7—C6—N2	120.73 (12)	C20—C25—H25	120.3
C11—C6—N2	118.75 (12)	C24—C25—C20	119.39 (14)
C6—C7—H7	120.2	C24—C25—H25	120.3
C8—C7—C6	119.67 (13)	N6—C26—C27	105.65 (11)
C8—C7—H7	120.2	O3—C26—C27	131.05 (13)
C7—C8—C9	120.20 (14)	O3—C26—N6	123.23 (12)
C7—C8—H8	119.9	C26—C27—C13	120.43 (11)
C9—C8—H8	119.9	C28—C27—C13	132.34 (12)
C8—C9—H9	120.0	C28—C27—C26	107.22 (11)
C10—C9—C8	119.95 (14)	C27—C28—C29	131.96 (12)
C10—C9—H9	120.0	C27—C28—N5	110.67 (11)
C9—C10—C11	120.53 (14)	N5—C28—C29	117.30 (12)
C9—C10—H10	119.7	C28—C29—H29A	109.5
C11—C10—H10	119.7	C28—C29—H29B	109.5
C6—C11—H11	120.5	C28—C29—H29C	109.5
C10—C11—C6	119.07 (13)	H29A—C29—H29B	109.5
C10—C11—H11	120.5	H29A—C29—H29C	109.5
C3—N1—C5	120.26 (12)	H29B—C29—H29C	109.5
C3—N1—N2	105.75 (11)	H30A—C30—H30B	109.5
N2—N1—C5	116.26 (11)	H30A—C30—H30C	109.5
C1—N2—C6	125.95 (12)	H30B—C30—H30C	109.5
C1—N2—N1	109.88 (11)	N5—C30—H30A	109.5
N1—N2—C6	120.01 (11)	N5—C30—H30B	109.5
C13—C12—H12A	109.5	N5—C30—H30C	109.5
C13—C12—H12B	109.5	C32—C31—N6	118.45 (12)
C13—C12—H12C	109.5	C36—C31—C32	120.83 (12)
H12A—C12—H12B	109.5	C36—C31—N6	120.72 (12)
H12A—C12—H12C	109.5	C31—C32—H32	120.5
H12B—C12—H12C	109.5	C33—C32—C31	119.03 (13)
C12—C13—C14	107.43 (11)	C33—C32—H32	120.5
C16—C13—C12	107.15 (10)	C32—C33—H33	119.8
C16—C13—C14	110.26 (11)	C34—C33—C32	120.48 (13)
C27—C13—C12	113.43 (11)	C34—C33—H33	119.8
C27—C13—C14	106.50 (10)	C33—C34—H34	120.1
C27—C13—C16	111.99 (10)	C35—C34—C33	119.89 (13)
C13—C14—H14A	109.5	C35—C34—H34	120.1
C13—C14—H14B	109.5	C34—C35—H35	119.8
C13—C14—H14C	109.5	C36—C35—C34	120.35 (13)
H14A—C14—H14B	109.5	C36—C35—H35	119.8
H14A—C14—H14C	109.5	C31—C36—H36	120.3
H14B—C14—H14C	109.5	C35—C36—C31	119.41 (13)
N4—C15—C16	105.96 (11)	C35—C36—H36	120.3
O2—C15—C16	130.79 (12)	C17—N3—C19	119.52 (11)
O2—C15—N4	123.24 (12)	C17—N3—N4	105.74 (10)
C15—C16—C13	121.04 (11)	N4—N3—C19	114.42 (10)
C17—C16—C13	131.90 (12)	C15—N4—C20	125.25 (11)
C17—C16—C15	106.99 (11)	C15—N4—N3	109.50 (10)
C16—C17—C18	132.34 (13)	N3—N4—C20	119.68 (11)
C16—C17—N3	111.14 (11)	C28—N5—C30	121.43 (11)
N3—C17—C18	116.50 (11)	C28—N5—N6	105.82 (10)
C17—C18—H18A	109.5	N6—N5—C30	113.40 (10)
C17—C18—H18B	109.5	C26—N6—C31	125.14 (11)
C17—C18—H18C	109.5	C26—N6—N5	110.14 (10)
H18A—C18—H18B	109.5	N5—N6—C31	119.77 (10)
H18A—C18—H18C	109.5		
			
N2—C1—C2—C3	−1.74 (15)	C23—C22—C21—C20	−2.3 (2)
O1—C1—C2—C3	177.25 (15)	C21—C22—C23—C24	0.9 (2)
C4—C3—C2—C1	175.98 (14)	C25—C24—C23—C22	1.3 (2)
N1—C3—C2—C1	−3.07 (16)	C23—C24—C25—C20	−2.1 (2)
C11—C6—C7—C8	2.6 (2)	C13—C27—C26—N6	−178.31 (11)
N2—C6—C7—C8	−176.75 (12)	C13—C27—C26—O3	4.7 (2)
C9—C8—C7—C6	−1.5 (2)	C28—C27—C26—N6	1.73 (14)
C7—C8—C9—C10	−0.9 (2)	C28—C27—C26—O3	−175.24 (14)
C11—C10—C9—C8	2.1 (2)	C29—C28—C27—C13	−0.2 (2)
C10—C11—C6—C7	−1.4 (2)	C29—C28—C27—C26	179.77 (13)
C10—C11—C6—N2	177.97 (12)	N5—C28—C27—C13	−177.14 (12)
C6—C11—C10—C9	−1.0 (2)	N5—C28—C27—C26	2.81 (15)
C5—N1—C3—C2	140.75 (13)	C36—C31—C32—C33	0.4 (2)
C5—N1—C3—C4	−38.41 (19)	N6—C31—C32—C33	−179.81 (12)
N2—N1—C3—C2	6.58 (15)	C34—C33—C32—C31	0.0 (2)
N2—N1—C3—C4	−172.58 (12)	C32—C33—C34—C35	−0.3 (2)
C6—N2—C1—C2	162.62 (12)	C36—C35—C34—C33	0.1 (2)
C6—N2—C1—O1	−16.5 (2)	C35—C36—C31—C32	−0.6 (2)
N1—N2—C1—C2	5.83 (14)	C35—C36—C31—N6	179.63 (12)
N1—N2—C1—O1	−173.27 (12)	C31—C36—C35—C34	0.3 (2)
C1—N2—C6—C7	−127.36 (14)	C19—N3—C17—C16	139.18 (12)
C1—N2—C6—C11	53.23 (18)	C19—N3—C17—C18	−39.15 (17)
N1—N2—C6—C7	27.30 (18)	N4—N3—C17—C16	8.40 (14)
N1—N2—C6—C11	−152.11 (12)	N4—N3—C17—C18	−169.93 (11)
C1—N2—N1—C3	−7.69 (14)	C15—N4—C20—C21	−138.74 (14)
C1—N2—N1—C5	−143.99 (12)	C15—N4—C20—C25	41.86 (18)
C6—N2—N1—C3	−166.08 (11)	N3—N4—C20—C21	12.09 (18)
C6—N2—N1—C5	57.62 (16)	N3—N4—C20—C25	−167.32 (11)
C12—C13—C16—C15	−67.73 (15)	C20—N4—C15—C16	157.61 (12)
C12—C13—C16—C17	108.90 (16)	C20—N4—C15—O2	−21.5 (2)
C14—C13—C16—C15	48.90 (16)	N3—N4—C15—C16	4.31 (14)
C14—C13—C16—C17	−134.48 (15)	N3—N4—C15—O2	−174.79 (12)
C27—C13—C16—C15	167.29 (11)	C15—N4—N3—C17	−7.68 (14)
C27—C13—C16—C17	−16.1 (2)	C15—N4—N3—C19	−141.33 (12)
C12—C13—C27—C26	175.73 (11)	C20—N4—N3—C17	−162.70 (11)
C12—C13—C27—C28	−4.3 (2)	C20—N4—N3—C19	63.66 (15)
C14—C13—C27—C26	57.76 (15)	C30—N5—C28—C27	−137.23 (12)
C14—C13—C27—C28	−122.29 (15)	C30—N5—C28—C29	45.31 (17)
C16—C13—C27—C26	−62.84 (15)	N6—N5—C28—C27	−6.17 (14)
C16—C13—C27—C28	117.11 (15)	N6—N5—C28—C29	176.37 (11)
N4—C15—C16—C13	178.23 (11)	C28—N5—N6—C26	7.30 (13)
N4—C15—C16—C17	0.86 (14)	C28—N5—N6—C31	163.66 (11)
O2—C15—C16—C13	−2.8 (2)	C30—N5—N6—C26	142.79 (11)
O2—C15—C16—C17	179.86 (14)	C30—N5—N6—C31	−60.86 (15)
C18—C17—C16—C13	−4.8 (3)	C31—N6—C26—C27	−160.42 (11)
C18—C17—C16—C15	172.16 (14)	C31—N6—C26—O3	16.9 (2)
N3—C17—C16—C13	177.21 (12)	N5—N6—C26—C27	−5.62 (13)
N3—C17—C16—C15	−5.81 (15)	N5—N6—C26—O3	171.66 (12)
C25—C20—C21—C22	1.5 (2)	C26—N6—C31—C32	−57.86 (17)
N4—C20—C21—C22	−177.89 (12)	C26—N6—C31—C36	121.90 (14)
C21—C20—C25—C24	0.7 (2)	N5—N6—C31—C32	149.55 (12)
N4—C20—C25—C24	−179.91 (12)	N5—N6—C31—C36	−30.68 (17)

### Hydrogen-bond geometry (Å, º)

**Table:**

*D*—H···*A*	*D*—H	H···*A*	*D*···*A*	*D*—H···*A*
C2—H2···O1^i^	0.95	2.50	3.2949 (17)	142
C5—H5*C*···O2	0.98	2.44	3.3930 (17)	163
C10—H10···O3^ii^	0.95	2.53	3.4670 (18)	171
C14—H14*A*···O3	0.98	2.45	3.1228 (17)	126
C14—H14*B*···O2	0.98	2.34	3.0275 (17)	126
C21—H21···O1^iii^	0.95	2.49	3.3428 (18)	150
C25—H25···O2	0.95	2.37	2.8811 (17)	113
C29—H29*B*···O3^iv^	0.98	2.38	3.2956 (17)	155
C30—H30*A*···O3^iv^	0.98	2.32	3.3015 (16)	176

Symmetry codes: (i) −*x*+3/2, *y*−1/2, −*z*+1/2; (ii) *x*+1, *y*+1, *z*; (iii) *x*−1, *y*−1, *z*; (iv) *x*, *y*+1, *z*.
